# Hemipteran Mitochondrial Genomes: Features, Structures and Implications for Phylogeny

**DOI:** 10.3390/ijms160612382

**Published:** 2015-06-01

**Authors:** Yuan Wang, Jing Chen, Li-Yun Jiang, Ge-Xia Qiao

**Affiliations:** 1 Key Laboratory of Zoological Systematics and Evolution, Institute of Zoology, Chinese Academy of Sciences, Beijing 100101, China; E-Mails: wangyuan0330@163.com (Y.W.); chenjing@ioz.ac.cn (J.C.); jiangliyun@ioz.ac.cn (L.-Y.J.); 2 College of Life Sciences, University of Chinese Academy of Sciences, Beijing 100049, China

**Keywords:** Hemiptera, mitogenomes, rearrangement, phylogenetic relationships

## Abstract

The study of Hemipteran mitochondrial genomes (mitogenomes) began with the Chagas disease vector, *Triatoma dimidiata*, in 2001. At present, 90 complete Hemipteran mitogenomes have been sequenced and annotated. This review examines the history of Hemipteran mitogenomes research and summarizes the main features of them including genome organization, nucleotide composition, protein-coding genes, tRNAs and rRNAs, and non-coding regions. Special attention is given to the comparative analysis of repeat regions. Gene rearrangements are an additional data type for a few families, and most mitogenomes are arranged in the same order to the proposed ancestral insect. We also discuss and provide insights on the phylogenetic analyses of a variety of taxonomic levels. This review is expected to further expand our understanding of research in this field and serve as a valuable reference resource.

## 1. Introduction

Entomologists first suggested that Hemiptera (true bugs) and Homoptera (planthoppers, leafhoppers, cicadas, spittlebugs, aphids, psyllids, scales, and whiteflies) are two orders according to features of the wing [1]. In 1810, Latreille suggested combining Heteroptera and Homoptera as one order called Hemiptera (*s.l.*) [2]. The concept of Hemiptera (*s.l.*) has been widely accepted since 1969 to the present [3,4,5]; therefore, in this review, Hemiptera refers to Hemiptera (*s.l.*). As one major order of insects, Hemiptera is the largest group of the hemimetabolous insects [6], including more than 50,000 described species [7]. They are small sap-sucking insects with body-sizes from 1 mm (0.04 in) to approximately 15 cm (6 in).

There is great variety within the order Hemiptera, more commonly known as bugs. Hemipterans have evolved an extraordinary range of body forms and lifestyles: some live on land, some live in water, some feed on plants and others are voracious carnivores or scavengers. Therefore, many species of Hemiptera are significant pests of crops and gardens. Some, as many species of aphid, cause direct damage to crop hosts and often kill the entire plants. Additionally, some delphacids cause considerable damage to grain production and have been identified as one cause of rice famine in several Asian countries [8]. Moreover, many species of Hemiptera are vectors of viruses and diseases. For example, *Triatoma dimidiata* is the vector of Chagas disease, a predominantly chronic disease affecting millions of people [9].

Based on the history of Hemipteran phylogeny research, we propose two controversial questions. First, how many suborders does Hemiptera include? Traditionally, Hemiptera comprised three major groups (including four suborders): Sternorrhyncha (aphids, scale bugs, whiteflies, and psyllids), Auchenorrhyncha (planthoppers, leafhoppers, spittlebugs, and cicadas), and Heteroptera (true bugs, including Coleorrhyncha) [10]. Previous morphological studies suggested that Fulgoromorpha and Cicadomorpha formed Auchenorrhyncha, and that Auchenorrhyncha is more closely related to Coleorrhyncha and Sternorrhyncha than to Heteroptera [11]. However, additional molecular and morphological evidence has challenged the monophyly of Auchenorrhyncha (summarized by [12]). The second question, what are the relationships of these suborders that have confused entomologists for many years? Cobben suggested that both Heteroptera and Fulgoromorpha form the sister clade to (Sternorrhyncha, Cicadomorpha) according to a cladistic study of morphological traits [13]. Hamilton examined the phylogenetic affiliations using features of the head and mouthparts and suggested (Fulgoromorpha, (Sternorrhyncha, Cicadomorpha)) was the sister group to the clade (Coleorrhyncha, Heteroptera) [14]. However, it has been argued that Coleorrhyncha and Heteroptera do not have an immediate common ancestor and have descended independently from separate lineages [15]. Hence, the phylogenetic relationships among the higher-level hemipteran lineages remain unclear.

Since the first insect mitogenome was published in 1985 [16], there has been a rapid accumulation of sequenced insect genomes, with representatives from all orders now available [17]. Insect mitogenomes are small, double stranded, circular DNA molecules, ranging in size from 14 to 19 kb. The mitogenome is composed of thirty-seven genes (13 protein-coding, 22 transfer RNA, and 2 ribosomal RNA genes), and contains a control region (A + T-rich region) that is thought to play a role in the initiation of transcription and replication, and is a source of length variation in the genome [18]. Particularly, mitogenome sequences can provide even more genetic information and are increasingly being utilized in insect identification, biogeographic and phylogenetic studies [19,20,21].

Here, we utilize all the mitogenomes of Hemiptera to analyze their features on the genome level and summarize the rearrangement events for the first time. In addition, all available complete mitogenomes of Hemiptera were used to reconstruct and discuss the phylogeny relationships of this order.

## 2. Mitogenomes of Hemiptera

**Figure 1:**
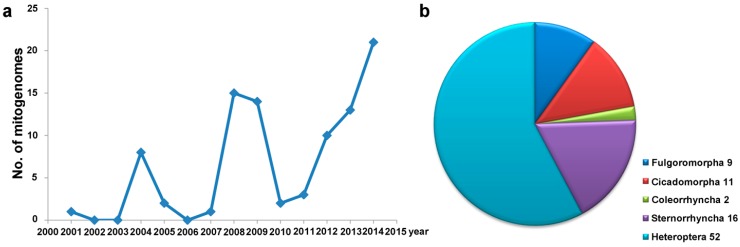
Accumulation of mitogenome data from Hemiptera. (**a**) The number of species sequenced in each year is represented by the blue line; (**b**) The number of species sequenced in each suborder is represented by the different pieces of the pie graph.

*Triatoma dimidiata*, the vector of Chagas disease, was the first published mitogenome of Hemipterain 2001 [9]. The sequencing history of hemipteran mitogenomes was shown (Figure 1a). There are two peaks during the past 14 years. Three years after the publication of the mitogenome of *Triatoma dimidiata*, Thao *et al.* [22] reported the complete nucleotide sequence of the mitogenomes of six species of whiteflies, one psyllid and one aphid from the suborder Sternorrhyncha. Four species of whiteflies had variations in gene order that were very different from the proposed insect ancestor (*Drosophila yakuba*) [16]. Subsequently, a number of studies have already proved that the rearrangements were more likely to happen in the mitogenomes of whiteflies than other insects of Hemiptera [22,23,24,25]. In 2008, Bu’s group obtained 10 complete and five nearly complete mitogenomes of Heteroptera [23] and they reported the first comparative mitogenome analysis of one suborder of Hemiptera and the phylogenetic relationships of Heteroptera [23]. With the development of PCR technology and the use of next-generation sequencing strategies [26,27,28], many complete mitogenome sequences of Hemiptera have been obtained and more will be sequenced (Figure 1a).

Figure 1b and Table 1 summarize the mitogenomes of Hemiptera from the first report to the present. The total 90 complete mitogenomes can be divided into five parts by different suborders (Figure 1b) (according to the five suborder system) [12]. Heteroptera has the highest species richness of Hemiptera [29], and more than a half of the 90 complete mitogenomes are from this suborder. Coleorrhyncha, small bugs with a cryptic lifestyle, possess a mixture of cicadomorphan and bug-like characters [30], and represent a separate suborder within Hemiptera. This suborder includes a single extant family, Peloridiidae, which is currently distributed only in Patagonia and on the Australian continent [31]. Only two complete mitogenomes of Peloridiidae have been reported to date [12,32], as representatives of Coleorrhyncha. Meanwhile, the provenances and GenBank numbers of these mitogenomes were detailed set out (Table 1). Most of them as the representatives of different taxa in Hemiptera were published for the first time [12,22,23,33].

**Table 1:** All available complete mitogenomes of Hemiptera.

Suborder	Family	Species	GenBank No.	Reference
Cicadomorpha	Aphrophoridae	*Philaenus spumarius*	NC_005944	[34]
	Cercopidae	*Abidama producta*	NC_015799	[35]
	Cercopidae	*Aeneolamia contigua*	NC_025495	[35]
	Cercopidae	*Callitetix braconoides*	NC_025497	[35]
	Cercopidae	*Callitetix versicolor*	EU725832	[35]
	Cercopidae	*Callitettix biformis*	NC_025496	[35]
	Cercopidae	*Paphnutius ruficeps*	NC_021100	[36]
	Cicadellidae	*Empoasca vitis*	NC_024838	[37]
	Cicadellidae	*Homalodisca coagulata*	AY875213	-
	Cicadellidae	*Homalodisca vitripennis*	NC_006899	*
	Membracidae	*Leptobelus gazella*	NC_023219	*
Coleorrhyncha	Peloridiidae	*Hackeriella veitchi*	GQ884145	[12]
	Peloridiidae	*Hemiodoecus leai*	NC_025329	[32]
Fulgoromorpha	Delphacidae	*Laodelphax striatella*	JX880068	[27]
	Delphacidae	*Laodelphax striatellus*	NC_013706	[38]
	Delphacidae	*Nilaparvata lugens*	NC_021748	[27]
	Delphacidae	*Nilaparvata muiri*	NC_024627	-
	Flatidae	*Geisha distinctissima*	NC_012617	[39]
	Fulgoridae	*Laternaria candelaria*	NC_019576	[40]
	Fulgoridae	*Lycorma delicatula*	NC_012835	[19]
	Issidae	*Sivaloka damnosa*	NC_014286	[41]
	Ricaniidae	*Ricania marginalis*	JN242415	[40]
Heteroptera	Alydidae	*Riptortus pedestris*	NC_012462	[23]
	Anthocoridae	*Orius niger*	NC_012429	[23]
	Anthocoridae	*Orius sauteri*	NC_024583	[42]
	Aradidae	*Aradacanthia heissi*	HQ441233	[43]
	Aradidae	*Brachyrhynchus hsiaoi*	NC_022670	[44]
	Aradidae	*Neuroctenus parus*	NC_012459	[23]
	Berytidae	*Yemmalysus parallelus*	NC_012464	[23]
	Colobathristidae	*Phaenacantha marcida*	NC_012460	[23]
	Coreidae	*Hydaropsis longirostris*	NC_012456	[23]
	Cydnidae	*Macroscytus gibbulus*	EU427338	[23]
	Enicocephalidae	*Stenopirates sp.*	NC_016017	[45]
	Gelastocoridae	*Nerthra indica*	NC_012838	[19]
	Geocoridae	*Geocoris pallidipennis*	NC_012424	[23]
	Gerridae	*Aquarius paludum*	NC_012841	[19]
	Hydrometridae	*Hydrometra greeni*	NC_012842	[19]
	Largidae	*Physopelta gutta*	NC_012432	[23]
	Lygaeidae	*Kleidocerys resedae*	KJ584365	[46]
Heteroptera	Malcidae	*Chauliops fallax*	NC_020772	[47]
	Malcidae	*Malcus inconspicuus*	NC_012458	[23]
	Miridae	*Adelphocoris fasciaticollis*	NC_023796	[48]
	Miridae	*Apolygus lucorum*	NC_023083	[49]
	Miridae	*Lygus lineolaris*	EU401991	-
	Miridae	*Nesidiocoris tenuis*	NC_022677	[50]
	Nabidae	*Alloeorhynchus bakeri*	HM235722	[51]
	Nabidae	*Gorpis annulatus*	NC_019595	[24]
	Nabidae	*Gorpis humeralis*	NC_019593	[24]
	Nabidae	*Nabis apicalis*	NC_019594	[24]
	Naucoridae	*Ilyocoris cimicoides*	NC_012845	[19]
	Nepidae	*Laccotrephes robustus*	NC_012817	[19]
	Notonectidae	*Enithares tibialis*	NC_012819	[19]
	Ochteridae	*Ochterus marginatus*	NC_012820	[19]
	Pentatomidae	*Dolycoris baccarum*	NC_020373	[52]
	Pentatomidae	*Halyomorpha halys*	NC_013272	[53]
	Pentatomidae	*Nezara viridula*	NC_011755	[23]
	Plataspidae	*Coptosoma bifaria*	NC_012449	[23]
	Plataspidae	*megacopta cribraria*	NC_015342	*
	Pleidae	*Paraplea frontalis*	NC_012822	[19]
	Pyrrhocoridae	*Dysdercus cingulatus*	NC_012421	[23]
	Reduviidae	*Agriosphodrus dohrni*	NC_015842	[54]
	Reduviidae	*Brontostoma colossus*	NC_024745	[28]
	Reduviidae	*Oncocephalus breviscutum*	NC_022816	[55]
	Reduviidae	*Peirates arcuatus*	NC_024264	[56]
	Reduviidae	*Sirthenea flavipes*	NC_020143	[57]
	Reduviidae	*Triatoma dimidiata*	NC_002609	[9]
	Reduviidae	*Valentia hoffmanni*	NC_012823	[19]
	Rhopalidae	*Aeschyntelus notatus*	NC_012446	[23]
	Rhopalidae	*Stictopleurus subviridis*	NC_012888	-
	Saldidae	*Saldula arsenjevi*	NC_012463	[23]
	Tessaratomidae	*Eusthenes cupreus*	NC_022449	[58]
	Tingidae	*Corythucha ciliata*	NC_022922	[59]
	Tingidae	*Pseudacysta perseae*	NC_025299	*
	Urostylididae	*Urochela quadrinotata*	NC_020144	[60]
Sternorrhyncha	Aleyrodidae	*Aleurochiton aceris*	NC_006160	[22]
	Aleyrodidae	*Aleurodicus dugesii*	NC_005939	[22]
	Aleyrodidae	*Bemisia afer*	NC_024056	[25]
	Aleyrodidae	*Bemisia tabaci*	NC_006279	[22]
	Aleyrodidae	*Neomaskellia andropogonis*	NC_006159	[22]
	Aleyrodidae	*Tetraleurodes acaciae*	NC_006292	[22]
	Aleyrodidae	*Trialeurodes vaporariorum*	NC_006280	[22]
	Aphididae	*Acyrthosiphon pisum*	NC_011594	*
	Aphididae	*Aphis gossypii*	NC_024581	[61]
	Aphididae	*Cavariella salicicola*	NC_022682	[62]
	Aphididae	*Cervaphis quercus*	NC_024926	[33]
	Aphididae	*Diuraphis noxia*	NC_022727	[63]
	Aphididae	*Schizaphis graminum*	NC_006158	[22]
	Aphididae	*Sitobion avenae*	NC_024683	[64]
	Psyllidae	*Pachypsylla venusta*	NC_006157	[22]
	Psyllidae	*Paratrioza sinica*	NC_024577	[65]

Legend: “-” refer to direct submission; “*” refers to submitted the data and not a published paper.

## 3. Features of Hemipteran Mitogenomes

### 3.1. Genome Organization

**Figure 2:**
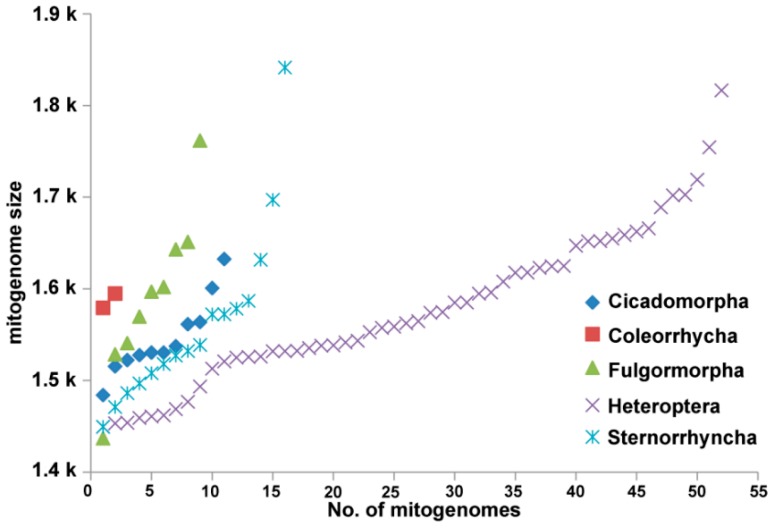
The size variation of mitogenomes from Hemiptera.

The mitogenome sizes of Hemiptera range from 14,371 bp (*Nilaparvata muiri*) to 18,414 bp (*Trialeurodes vaporariorum*) and have an average value of 15,733 bp (Figure 2). The size changes of five suborders are also shown (Figure 2). The size variation is mainly attributed to the non-coding regions, especially the control regions and repeat regions in some groups (such as the control regions of the true water bugs [23] and the repeat regions of aphids [62]).

Most of the mitogenomes (76/90) resemble that of the known ancestral species (*D. yakuba* [16]) in structural organization and composition with 13 protein coding genes (PCGs), 22 transfer RNAs (tRNAs), and 2 ribosomal RNAs (rRNAs). The remaining mitogenomes differ only in the number of tRNAs, most likely due to gene deletion events. For example, *Neomaskellia andropogonis* (Sternorrhyncha) contains only 18 tRNAs [22].

### 3.2. Nucleotide Composition

**Figure 3:**
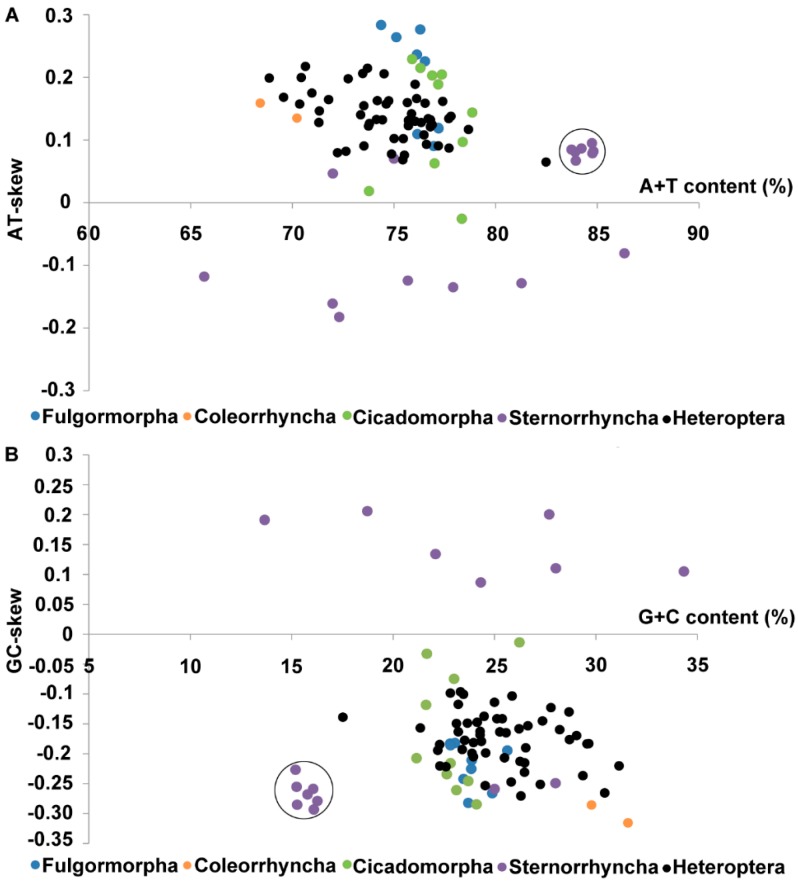
Nucleotide composition across 90 complete hemipteran mitogenomes. (**A**) A + T content and AT skew; (**B**) G+C content and GC skew. Dots in the cycle represent values for all the seven aphid mitogenomes.

The A%, T%, C% and G% values and the AT and GC skews were calculated for all available complete mitogenomes of Hemiptera species (Figure 3). Interestingly, the lowest and the highest A + T contents of the hemipteran mitogenomes were found in the suborder Sternorrhyncha (65.67% in *Bemisia afer* and 86.33% in *Aleurodicus dugesii*). Species from the suborders Fulgoromorpha, Coleorrhyncha and Heteroptera were all A and C skewed. This was also the case for the species of Cicadomorpha, except for *Empoasca vitis*. For the suborder Sternorrhyncha, nine species were A and C skewed, including all aphid species. This discovery of all aphid species forming a cluster is similar to the results of previous studies (cycle in Figure 3 [62,63]). In contrast, the seven other Sternorrhynchan species (whiteflies), which had highly rearranged gene orders [22,25], were G and T skewed.

### 3.3. Protein-Coding Genes

All PCGs in the majority of hemipteran mitogenomes were initiated with familiar triplet initiation codons (as shown in the invertebrate mitochondrial genetic code table), including the commonly used ATN and some special couplet codons. For instance, in the suborder Coleorrhyncha, *cox1* starts with CGA in *Xenophyes cascus* and with TCG in *Hackeriella veitchi* [12]. Furthermore, the tetranucleotide initiation codons were also found in hemipteran mitogenomes; such as in Cydnidae where *nad2* was supposed to be initiated with an atypical initiation codon, ATCA [23]. In fact, atypical initiation codons are not rare in other insects; for example, the tetranucleotide TTAG is the initiation codon for *cox1* of *Bombyx mori* (Lepidoptera: Bombycidae) [66]. Most PCGs stopped with TAA/TAG termination codons or truncated termination codons (TA or T) that are presumed to be completed via posttranscriptional polyadenylation [67].

**Figure 4:**
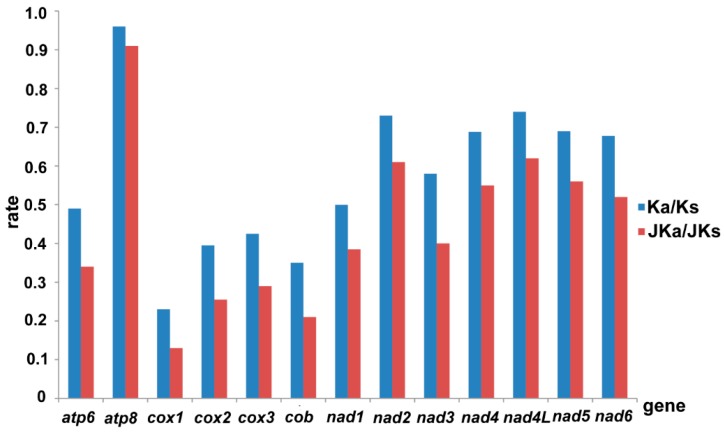
Evolutionary rates of protein-coding genes in hemipteran mitogenomes. The blue bar indicates the gene’s Ka/Ks, and the red bar indicates the Jukes-Cantor adjusting data.

In view of the evolutionary forces acting on the mitochondrial PCGs of hemipteran species, the average rate of non-synonymous substitutions (Ka), the average rate of synonymous substitutions (Ks), the average ratio of Ka/Ks, and the Jukes-Cantor adjusted Ka/Ks (JKa/JKs) were calculated for each PCG, respectively [68]. The results showed that *atp8* had the highest evolutionary rate, followed by *nad2*, while *cox1* appeared to be the lowest (Figure 4). Notably, the ratio of Ka/Ks for each PCG was below 1, indicating that these genes are evolving under purifying selection. The uniformly low values of the Ka/Ks and JKa/JKs ratios for *cox1–3* and *cob* indicate strong evolutionary constraints in cytochrome c oxidase [69] and also suggest a strong purifying selection in the species of Hemiptera. Therefore, a DNA barcoding approach based on *cox1* sequence diversity has been utilized for identification of closely related species [70]. Similarly, *cob* and *cox2* with relatively slow rates may also be candidate barcoding markers [24,62]. By contrast, due to the highest divergence, *atp8* and *nad2* can be used as an effective molecular marker to analyze intraspecific relationships and reveal relationships between populations within the same hemipteran species. This result is highly consistent with previous findings in most metazoans [71].

### 3.4. tRNAs and rRNAs

All 22 tRNA coding genes usually were found in the mitogenomes of Hemiptera and the tRNAs were between 60 and 75 bp in length. The anticodon nucleotides for the corresponding tRNA genes are identical to those of other available arthropod mitogenomes [66,72]. All tRNA genes had the typical clover-leaf structure with one exception: *trnS*(*AGN*), in which the dihydrouridine arm formed a simple loop (as in some other metazoan species, including most insects [66,72,73].

The arrangements of both *rrnL* and *rrnS* in the hemipteran mitogenomes are commonly conserved, and are generally located between *trnL*(*CUN*) and *trnV*, and between *trnV* and the control region. The lengths of *rrnL* and *rrnS* are determined to be 1192–1260 and 711–766 bp, respectively. These lengths are similar to those of other orders of Insecta [16,66,72,73].

### 3.5. Non-Coding Regions

There are some non-coding (NC) regions interspersed throughout the hemipteran mitogenomes, thus the mitogenomes of Hemiptera displayed a moderate size variation. Four distinct large NC regions were identified in the following gene pairs of hemipteran mitogenomes: *trnI*-*trnQ*, *trnS*-*nad1*, *trnE*-*trnF* and *rrnS*-*trnI*. The region located between *rrnS* and *trnI*, coincided with the A + T-rich region, also called the control region, including the origin of replication and promoters for transcription initiation [16,74,75]. Tandem repeats were detected in the remaining three regions, and named repeat regions.

#### 3.5.1. Control Region

**Figure 5:**
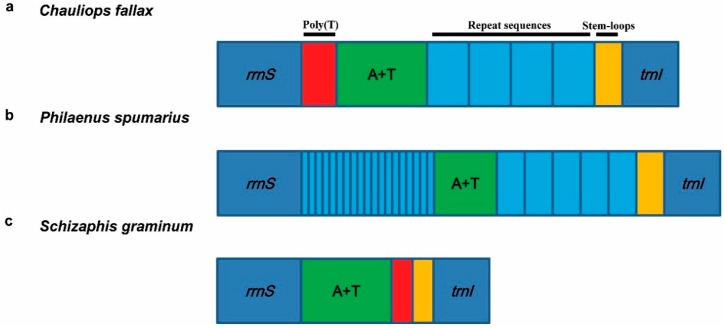
Control regions of mitogenomes from some representative species of Hemiptera. (**a**) the control region of *Chauliops fallax* includes four parts; (**b**) the control region of *Philaenus spumarius* includes two fragments of tandem repeat sequences; (**c**) the control region of *Schizaphis graminum* includes three parts without repeat sequences.

Most control regions of hemipteran mitogenomes were longer than 1 kb, with high rates of nucleotide substitution and indels, and a variable number of tandem repeats. Generally, one control region of the hemipteran mitogenome includes four parts without order: tandem repeat sequences, sequences of poly(T) stretch, a subregion with high A + T content, and stem-loop structures (for example, *Chauliops fallax* Figure 5a). This feature of the control region was summarized by Cook for arthropods [76]. There are some interesting exceptions in the hemipteran mitogenomes. For example, in some species of Cicadomorpha (*Philaenus spumarius*) [34], Fulgoromorpha (*Geisha distinctissima*, *Sivaloka damnosa*, *Laodelphax striatella* and *Laodelphax striatellus*) [27,38,39,41] and Heteroptera (*Alloeorhynchus bakeri*) [51], two fragments of tandem repeat sequences insert into the control region separately (for example, *Philaenus spumarius* Figure 5b). A few of the control regions of hemipteran species did not contain all four parts (for example, *Schizaphis graminum* Figure 5c) [22]. The conserved sequences, stem-loop structures and tandem repeat sequences found in the present study can provide useful information for research of the phylogeny of specific groups [34,35,45,47,62]. For example, in the systematic research of Aphidinae, the phylogenetic tree based on PCGs is similar to the clusters of the stem-loop structures [62]. Another interesting question is how functionality is retained under such great variations in both length and sequence. Considering the high nucleotide substitution rate, both the secondary structures and the conserved segments might be key clues in determining the function of the control region.

#### 3.5.2. Repeat Region

**Figure 6:**
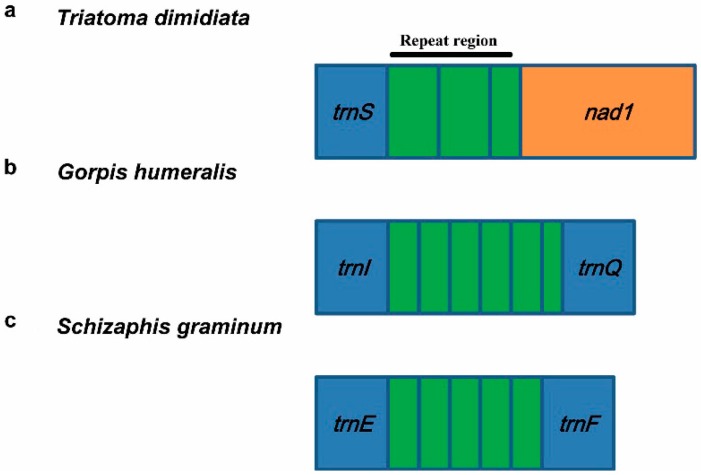
Repeat regions of mitogenomes from some representative species of Hemiptera. (**a**) the repeat region of *Triatoma dimidiata* (Hemiptera: Heteroptera: Reduviidae); (**b**) the repeat region of *Gorpis humeralis* (Hemiptera: Heteroptera: Nabidae); (**c**) the repeat region of *Schizaphis graminum* (Hemiptera: Sternorrhyncha: Aphididae).

In general, the NC regions of an insect mitogenome consist of a control region and short intergenic spacers. However, some special species of Hemiptera include one repeat region (Figure 6). These repeat regions mainly are located into different positions (*trnE*-*trnF*, *trnI*-*trnQ* and *trnS*-*nad1*) in three families (Aphididae, Nabidae and Reduviidae), and differ in repeat unit sequence and copy number, suggesting that they are highly species-specific (Table 2). These repeat regions are not similar to any known sequences in GenBank. We speculate that this region, full of tandem repeats, has a function similar to the intergenic spacer in *Apis mellifera* that is thought to be another origin of replication [77].

## 4. Hemipteran Mitogenome Arrangements and Evolution

Within Insecta, the order of the mitochondrial genes is highly conserved and has led to the proposal of an ancestral gene order [16]. The majority of hemipteran families that have been sequenced possess this ancestral insect arrangement. Gene orders of Coleorrhyncha and Cicadomorpha are mostly conserved; however, a few families of Fulgoromorpha, Sternorrhyncha and Heteroptera show extreme rearrangement (Table 3). The three rearrangement types of gene movements, transposition, inversion, and inverse transposition [78], were all found in the hemipteran mitogenomes (Table 3). Two species in the superfamily Pyrrhocoroidea share the same gene order with the inversion of *trnT* and *trnP* [23]. Whiteflies (Aleyrodidae) are the group that is most likely to have rearrangements: *cox3*-*trnG*-*nad3* is inverse transposed into three different locations in the mitogenome [22]. In *Stenopirates* sp., the most striking features were the inversion of two tRNA genes (*trnT* and *trnP*) and the transpositions of five gene clusters (trn*T*-trn*P*-*nad6*, *cob*-*trnS*, *nad1*-*trnL*, *rrnL*-*trnV*-*rrnS* and control region) between *nad4L* and *trnI* [45]. The four hot spots of rearrangements are summarized: 1. upstream of *nad2*; 2. between *nad2* and *cox1*; 3. between *nad4L* and *nad1*; and 4. downstream of *rrnL* (Table 3).

**Table 2:** Repeat regions of hemipteran mitogenomes.

Species	Classification	Location	Repeat Number	Repeat Unit Size	Reference
*Agriosphodrus dohrni*	Heteroptera: Reduviidae	*trnS-nad1*	two and a partial	58 bp	[54]
*Triatoma dimidiata*	Heteroptera: Reduviidae	*trnS-nad1*	two and a partial	135 bp	[9]
*Gorpis annulatus*	Heteroptera: Nabidae	*trnS-nad1*	three and a partial	179 bp	[24]
*Gorpis humeralis*	Heteroptera: Nabidae	*trnS-nad1*	two and a partial	188 bp	[24]
*Gorpis humeralis*	Heteroptera: Nabidae	*trnI-trnQ*	five and a partial	244 bp	[24]
*Himacerus nodipes*	Heteroptera: Nabidae	*trnI-trnQ*	four	135 bp	[24]
*Acyrthosiphon pisum*	Sternorrhyncha: Aphididae	*trnE-trnF*	seven and a partial	203–206 bp	*
*Aphis gossypii*	Sternorrhyncha: Aphididae	*trnE-trnF*	four and a partial	196 bp	[61]
*Cavariella salicicola*	Sternorrhyncha: Aphididae	*trnE-trnF*	three	199 bp	[62]
*Diuraphis noxia*	Sternorrhyncha: Aphididae	*trnE-trnF*	three and a partial	194–195 bp	[63]
*Schizaphis graminum*	Sternorrhyncha: Aphididae	*trnE-trnF*	four and a partial	151–153 bp	[22]
*Sitobion avenae*	Sternorrhyncha: Aphididae	*trnE-trnF*	one and a partial	202 bp	[64]

Legend: “*” refers to submitted the data and not a published paper.

**Table 3:** Mitogenome rearrangements found in Hemiptera.

Classification	Species	Level	Rearrangement	Reference
Fulgoromorpha: Delphacidae	*Laodelphax striatella*	family	Inversion of *trnC* and *trnW*, inverse transposition: *trnT*-*trnP*-*nad6* → *nad6*-*trnP*-*trnT*	[27]
Fulgoromorpha: Delphacidae	*Laodelphax striatellus*	family	Inversion of *trnC* and *trnW*, transposition of *trnH*, and inverse transposition: *trnT*-*trnP*-*nad6* → *nad6*-*trnP*-*trnT*	[38]
Fulgoromorpha: Delphacidae	*Nilaparvata lugens*	family	Inversion of *trnC* and *trnW*, inverse transposition: *trnT*-*trnP*-*nad6* → *nad6*-*trnP*-*trnT*, and insertion two *trnC*	[27]
Heteroptera: Aradidae	*Aradacanthia heissi*	species	Inversion of *trnI* and *trnQ*, inversion of *trnC* and *trnW*	[43]
Heteroptera: Aradidae	*Brachyrhynchus hsiaoi*	genus	Inversion of *trnI* and *trnQ*	[44]
Heteroptera: Aradidae	*Neuroctenus parus*	genus	Inversion of *trnI* and *trnQ*	[23]
Heteroptera: Enicocephalidae	*Stenopirates* sp.	species	Inversion of *trnT* and *trnP*, inverse transposition: *trnT*-*trnP*-*nad6*-*cytB*-*trnS-nad1*-*trnL-rrnL*-*trnV*-*rrnS-*control region → *cytB*-*trnS-*control region*-rrnL*-*trnV*-*rrnS-nad1*-*trnL*-*trnP*-*trnT*-*nad6*	[45]
Heteroptera: Largidae	*Physopelta gutta*	superfamily	Inversion of *trnT* and *trnP*	[23]
Heteroptera: Pyrrhocoridae	*Dysdercus cingulatus*	superfamily	Inversion of *trnT* and *trnP*	[23]
Sternorrhyncha: Aleyrodidae	*Aleurochiton aceris*	genus	Inversion of *trnC* and *trnY*, inverse transposition: *cox3*-*trnG*-*nad3* → insertion the location *cob*-*nad1*	[22]
Sternorrhyncha: Aleyrodidae	*Aleurodicus dugesii*	genus	Inversion of *trnC* and *trnY*	[22]
Sternorrhyncha: Aleyrodidae	*Bemisia afer*	genus	Inversion of *trnC* and *trnY*, transposition of *trnQ*, and inverse transposition: *cox3*-*trnG*-*nad3* → insertion the location control region-*rrnS*	[25]
Sternorrhyncha: Aleyrodidae	*Bemisia tabaci*	genus	Inversion of *trnC* and *trnY*, transposition of *trnQ*, and inverse transposition: *cox3*-*trnG*-*nad3* → insertion the location control region-*rrnS*	[22]
Sternorrhyncha: Aleyrodidae	*Neomaskellia andropogonis*	genus	Transposition of *trnH* and *trnK*, and inverse transposition: *cox3*-*trnG*-*nad3* → insertion the location *rrnL*-*rrnS*	[22]
Sternorrhyncha: Aleyrodidae	*Tetraleurodes acaciae*	genus	Inversion of *trnC* and *trnY*, transposition of *trnQ* and *trnA*, and inverse transposition: *cox3*-*trnG*-*nad3* → insertion the location control region-*rrnS*	[22]
Sternorrhyncha: Aleyrodidae	*Trialeurodes vaporariorum*	genus	Inversion of *trnI* and *trnQ*, inversion of *trnC* and *trnY*, and transposition of *trnG*	[22]

Rearrangements of the mitogenomes are relatively rare events at the evolutionary scale [17]. Therefore, they can be powerful tool to delimit deep divergences among some insect lineages. The first discovery in this aspect of Hemiptera was found in whitefly in 2004. Thao *et al.* determined the complete mitogenomes of six whitefly species and their results indicated a clustering of whitefly species that corresponded to the gene arrangement types [22]. Then, in 2009, a comparison of gene orders and contents revealed that Hemiptera had three conserved gene blocks shared by all 20 species [53]. Gene orders and contents of both Heteroptera and Auchenorrhyncha (Cicadomorpha and Fulgoromorpha) were mostly conserved, whereas those of Sternorrhyncha showed extreme rearrangement [53]. However, compared with *Laodelphax striatella* [27], *Stenopirates* sp. [45] and *Aradacanthia heissi* [43], rearrangements in species of Fulgoromorpha and other true bugs seem to occur independently of family or species (Table 3). These results suggest that mitogenome orders might lack the resolution to deduce phylogenetic relationships among infraorders within Fulgoromorpha and Heteroptera.

## 5. Phylogenetic Inferences by Hemipteran Mitogenomes

**Figure 7:**
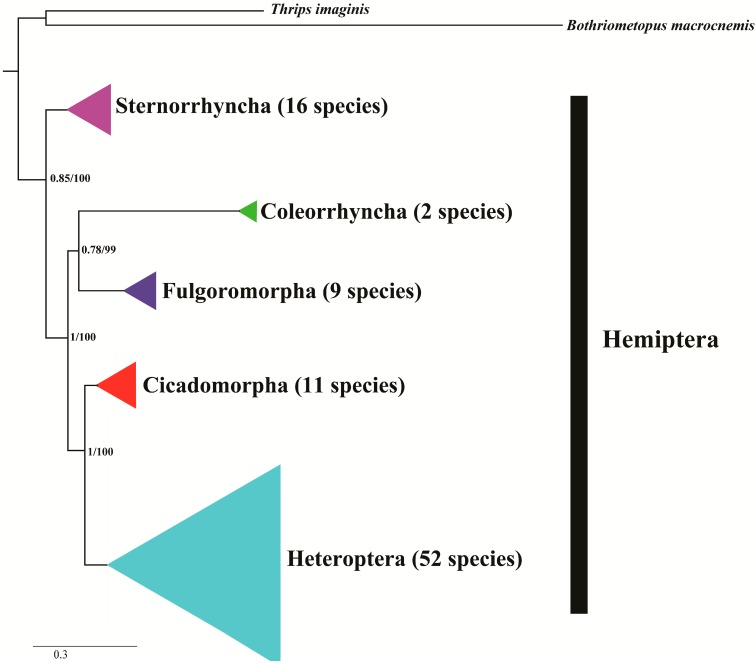
ML and BI Phylogenetic tree inferred from 90 hemipteran mitogenome sequences based on 13 PCGs. The node support values are the Bayesian posterior probabilities (BPP) and the bootstrap (BS) values.

As mentioned in the introduction, the phylogenic relationship of the Hemiptera has been controversial for many years and two questions remain unanswered. Here, we reviewed the research history of hemipteran phylogenetic relationships based on mitogenomes and combine our phylogenetic analyses to discuss the most reliable results. In 2009, a study clarified the relationships of the three phylogenetically controversial suborders, Auchenorrhyncha, Sternorrhyncha, and Heteroptera [53]. Heteroptera constituted a monophyletic group, and a sister relationship was proposed for Auchenorrhyncha and Sternorrhyncha [53]. However, only one species (Cicadomorpha: *Philaenus spumarius*) was chosen representing Auchenorrhyncha, and no taxa of Fulgoromorpha were discussed. Therefore, in the same year, Song and Liang [38] increased the samplings of taxa and proposed the inferred genealogical proximities of hemipteran lineages of (Heteroptera + (Cicadomorpha + (Fulgoromorpha + Sternorrhyncha))). In their research, Auchenorrhyncha was clearly separated into two parts, and Fulgoromorpha and Cicadomorpha were not a monophyletic group [38]. In fact, in their reports (in 2010 and 2012), the paraphyly of Auchenorrhyncha was also supported [40,41], and their phylogenetic reconstruction supported a sister relationship between Fulgoromorpha and Sternorrhyncha [40]. The suborder Coleorrhyncha (Hemiptera) has only one extant family, Peloridiidae, comprising 36 species in 17 genera [79]. Species of this group live in the wet mosses of South America (Chile, Argentina), New Zealand, New Caledonia and eastern Australia (from North Queensland to Tasmania) [80]. Complete or nearly complete mitogenomes of Peloridiidae were not obtained until 2013 [12]. Cui’s research was the first phylogenomic study of hemipterans with complete suborder samplings. Their results supported the paraphyly of Auchenorrhyncha and proposed the close relationship between Cicadomorpha and Heteroptera [12]. Meanwhile, our result displayed the similar result (Figure 7): Sternorrhyncha located as the basal suborder and Cicadomorpha and Heteroptera clustered as sister-group. Summarizing all these viewpoints, we can make three conclusions. First, the phylogenetic relationships among suborder-level hemipteran linages remain unclear by using mitogenome inference. Most viewpoints supported that Auchenorrhyncha is not a monophyletic group; Second, whether a monophyletic group or a sister-group to Cicadomorpha, the suborder Heteroptera is the most evolved group of Hemiptera; Third, the positions of other suborders remain confused and require further investigation.

Under the suborder taxa, the phylogenetic research also was involved. We summarized all the research results of these years (Table 4). All the phylogenetic issues of every taxonomic category were considered. For example, in the relationships among the intraorders of Heteroptera, Enicocephalomorpha was the most basal sister-group of the majority of Heteroptera [45]; the position of Cimicomorpha was unclear and it is possible that it is not a monophyletic group [45,47]; and Pentatomomorpha was the most evolved group of Heteroptera [45,47,49,54,60]. Regarding interfamily relationships, Hua *et al.* conducted phylogenomic studies on the mitogenomes of Pentatomomorpha [23] and Nepomorpha [19], and resolved some superfamily phylogenetic problems (Table 4). In Sternorrhyncha, the mitochondrial gene rearrangements among whiteflies corresponded to the phylogenetic tree [22]. The intrasubfamily relationships of Aphids (a group with special regions [33,63]), were also discussed [62] (Table 4).

**Table 4:** The phylogenetic analyses under the suborder taxa of Hemiptera.

Classification	Level	Viewpoint	Reference
Cicadomorpha: Cercopidae and Aphrophoridae	family	The monophyly of five Callitettixini species.	[35]
Sternorrhyncha: Aleyrodidae, whiteflies	genus	Four types of the mitochondrial gene rearrangements among whiteflies were corresponding to the branches of phylogenetic tree.	[22]
Sternorrhyncha: Aphididae, aphids	subfamily	Treat pterocommatines as members of Macrosiphini.	[62]
Heteroptera: Reduviidae	subfamily	The monophyly of Reduviidae and the Peiratinae presents a sister position to the Triatominae + (Salyavatinae + Harpactorinae).	[57]
Heteroptera: Pentatomomorpha	superfamily	The monophyly of Pentatomoidea, Pyrrhocoroidea, Lygaeoidea, and Coreoidea; Aradoidea and the Trichophora are sister groups.	[23]
Heteroptera: Nepomorpha	superfamily	Pleoidea is not a member of the Nepomorpha and Aphelocheiroidea should be grouped back into Naucoroidea.	[19]
Heteroptera: Nabidae	subfamily	Three tribes from two subfamilies of Nabidae.	[24]
Heteroptera	intraorder	The paraphyly of Cimicomorpha, and within Reduviidae, Harpactorinae is a sister group to the Salyavatinae + Triatominae.	[54]
Heteroptera	intraorder	The paraphyly of Cimicomorpha, and Reduviidae was paraphyletic with respect to Anthocoridae and Miridae.	[60]
Heteroptera	intraorder	The sister-relationship within the individual infraorders are supported for the Pentatomomorpha, Nepomorpha, Leptopodomorpha and Gerromorpha; *Stenopirates* sp. (Enicocephalomorpha) is the sister group to all the remaining Heteroptera.	[45]
Heteroptera	intraorder	Two Gerromorpha superfamilies were monophyletic in the basal position of these five infraorders. Within Cimicomorpha, Reduviidae was paraphyletic with respect to Anthocoridae and Miridae.	[47]
Heteroptera	intraorder	*Stenopirates* sp. was the sister group to all the remaining Heteroptera; the sister relationships within Nepomorpha and Gerromorpha.	[49]

In conclusion, the present study shows that mitogenomes may be good molecular markers for phylogenetic inference at a variety of taxonomic levels of Hemiptera (such as suborders, intraorders and families). However, some relationships have not been resolved based solely on mitogenomes. Nuclear genes evolve more slowly, and are effective for the analysis of deeper phylogenetic relationships. Moreover, some endosymbionts co-evolve with their hosts, and symbiont-derived data, in principle, could be used to reconstruct the evolutionary history of hosts [81]. So, with the development of sequencing technology, more available genetic resources are expected to provide more effective information of phylogenetic trees.

## 6. Experimental Section

### 6.1. Sampling

A total of 90 taxa were sampled in this study (Table 1). In the phylogeny analyses, the outgroups were sampled from Phthiraptera (*Bothriometopus macrocnemis*, GenBank accession number: NC_009983) and Thysanoptera (*Thrips imaginis*, GenBank accession number: NC_004371).

### 6.2. Analysis of Sequence Data

The nucleotide sequences of PCGs were translated based on the invertebrate mtDNA genetic code. A + T content were calculated using MEGA version 6.0 [82]. Strand asymmetry was calculated using the formulae AT skew = [A − T]/[A + T] and GC skew = [G − C]/[G + C], for the strand encoding the majority of the protein-coding genes. The software packages DnaSP 5.0 [83] was used to calculate the synonymous substitution rate (Ks) and the nonsynonymous substitution rate (Ka) for each PCG as well as Jukes-Cantor adjusted Ka/Ks (JKa/JKs).

### 6.3. Phylogenetic Analysis

Each of the 13 PCGs of all 92 species were aligned individually using MEGA v6.0 [82] with default parameters. Before alignments, the stop codons were all removed from those sequences. Maximum likelihood (ML) and Bayesian inference (BI) analyses were implemented by PHYML 3.0 [84] and MrBayes version 3.1.2 [85], respectively. Model selection was based on jModeltest v0.1.1 [86]. According to the AIC, the GTR + I + G model was optimal for analysis with nucleotide alignments. MrBayes version 3.1.2 and PHYML were employed to reconstruct the phylogenetic trees. In the ML analysis, the parameters were estimated during analysis and the node support values were assessed by bootstrap re-sampling (BP) calculated using 100 replicates. In Bayesian inference, runs of ten million generations were conducted. Trees were sampled every 1000 generations with a burn-in of 25%.

## 7. Conclusions and Perspectives

Generally, the complete mitogenomes of Hemiptera were 14–17K bp in size and encoded all 37 genes typical for insects. These genes were arranged in the same order as the proposed ancestral insect, except in a few particular species. Notably, the mitogenomes of three families possessed a large repeat region located at three different positions. We speculate that this region, full of tandem repeats, is another origin of replication. The mitogenomes have been successfully used to reconstruct the phylogenetic relationships within a variety of taxonomic levels of Hemiptera.

Future work should focus on four goals. First, the comparative genomics of different categories need more taxon samplings and more mitogenome sequences to further describe the comprehensive characteristics of Hemiptera mitogenomes; Second, the research of various populations and phylogeographic structures of hemipteran species based on mitogenomes require more mitogenome sequences about the same species or similar species; Third, the functional and evolutionary significance of different rearrangement types should be examined to open the view of the evolutionary dynamics of Hemiptera mitogenomes; Finally, phylogenetic inference with more resource data will provide greater insight into the evolution of Hemiptera.
